# Immune system of the inner ear as a novel therapeutic target for sensorineural hearing loss

**DOI:** 10.3389/fphar.2014.00205

**Published:** 2014-09-02

**Authors:** Takayuki Okano

**Affiliations:** Department of Otolaryngology, Head and Neck Surgery, Graduate School of Medicine, Kyoto UniversityKyoto, Japan

**Keywords:** resident macrophages, autoimmunity, corticosteroids, cell therapy, molecular targeted drugs

## Abstract

Sensorineural hearing loss (SNHL) is a common clinical condition resulting from dysfunction in one or more parts in the auditory pathway between the inner ear and auditory cortex. Despite the prevalence of SNHL, little is known about its etiopathology, although several mechanisms have been postulated including ischemia, viral infection or reactivation, and microtrauma. Immune-mediated inner ear disease has been introduced and accepted as one SNHL pathophysiology; it responds to immunosuppressive therapy and is one of the few reversible forms of bilateral SNHL. The concept of immune-mediated inner ear disease is straightforward and comprehensible, but criteria for clinical diagnosis and the precise mechanism of hearing loss have not been determined. Moreover, the therapeutic mechanisms of corticosteroids are unclear, leading to several misconceptions by both clinicians and investigators concerning corticosteroid therapy. This review addresses our current understanding of the immune system in the inner ear and its involvement in the pathophysiology in SNHL. Treatment of SNHL, including immune-mediated inner ear disorder, will be discussed with a focus on the immune mechanism and immunocompetent cells as therapeutic targets. Finally, possible interventions modulating the immune system in the inner ear to repair the tissue organization and improve hearing in patients with SNHL will be discussed. Tissue macrophages in the inner ear appear to be a potential target for modulating the immune response in the inner ear in the pathophysiology of SNHL.

## INTRODUCTION

Sensorineural hearing loss (SNHL) is a collection of common auditory disorders resulting from dysfunction of the inner ear, auditory nerve, or the auditory processing pathway in the central nervous system. SNHL comprises a wide variety of auditory disorders including sudden deafness, age-related hearing loss, noise-induced hearing loss, and Meniere’s disease. To date, very little of the SNHL pathophysiology is known because biopsy of the human inner ear is not feasible. Among the purposed mechanisms, immune-mediated SNHL has been introduced and accepted in the last three decades.

The inner ear has been thought of as an immune privileged organ for a long time. The cochlea has no lymphatic drainage, and the blood-labyrinth barrier is tightly controlled to separate the cochlear microenvironment from the circulation. In addition, the concentration of immunoglobulin in the cochlear fluid is 1/1,000 of the concentration in the cerebrospinal fluid (Harris and Ryan, 1984). McCabe (1979) introduced the clinical definition of autoimmune inner ear disease as rapidly progressive bilateral hearing loss that responds to corticosteroid and immunosuppressive therapy. Corticosteroids have been widely used as the first and only regimen to treat virtually all types of SNHL with sudden onset or rapid progression even before McCabe’s report. The anti-inflammatory and immune suppressive functions of corticosteroids led to their common use for hearing loss, especially when inflammation or an immunological disorder is suspected. Despite the common use of corticosteroids for inner ear disorders, our understanding of their role in the pathogenesis of reversible hearing loss remains limited. Steroid-responsive hearing loss does not always indicate an underlying inflammation or immune disorder in the inner ear (Trune and Canlon, 2012). Topical application of corticosteroids in the tympanic cavity has also been reported in patients unable to tolerate systemic treatment due to global adverse effects (Kakehata et al., 2006; Han et al., 2009), and the functional mechanisms of systemic and topical corticosteroid application supposedly differ. Therefore, a better understanding of the inner ear immunology and mechanisms of corticosteroids in the inner ear would enable development of a more sophisticated therapy for SNHL, including immune-mediated inner ear disease. In addition, alternative strategies of modulating immune activity without corticosteroids are desirable for treating certain types of SNHL.

In this review, we will discuss the characteristics and suspected pathophysiology of clinical hearing loss mediated by the immune system. Second, we will describe the current understanding of the inner ear immune system and will explore recent advances in both basic and clinical research of the mechanism of corticosteroid therapy in the inner ear. Finally, we will discuss current and potential SNHL therapies, including treatments targeting immune-mediated inner ear disease.

## PATHOPHYSIOLOGY OF SNHL FROM AN IMMUNOLOGICAL VIEWPOINT

The pathophysiology of organ-specific autoimmune disease is believed to be initiated by three primary mechanisms: (i) production of autoantibodies against tissue antigens, (ii) deposition of antigen–antibody complexes in tissue, and (iii) infiltration and destruction of tissue by specific cytotoxic T-cells. To date, the mechanisms of hearing loss in immune-mediated inner ear disease has yet to be determined, and none of the three described pathophysiology mechanisms have been reported in the human inner ear.

Immune-mediated inner ear disease was originally defined by McCabe (1979), who stated that idiopathic bilateral SNHL progresses to deafness over weeks or months, not hours, days, or years, and responds to corticosteroid and immunosuppressive therapy. The term of autoimmune inner ear disease refers to a pathology restricted to the inner ear. The time course of hearing loss distinguishes immune-mediated inner ear disease from sudden deafness or age-related hearing loss. Although this clinical entity is probably immune-mediated as immunosuppressive agents are effective, there is no direct evidence that the condition is autoimmune in etiology because diagnostic biopsy of the human inner ear is not feasible. Moreover, there are no uniformly accepted diagnostic criteria of immune-mediated inner ear disease. The presence of bilateral SNHL of at least 30 dB with evidence of progression in at least one ear on two serial audiograms performed less than 3 months apart is often used as case criteria (Moscicki et al., 1994). Fluctuations in hearing may occur, and immune-mediated disease is one of the few reversible causes of SNHL. Further study is still required to determine the pathophysiologic mechanisms underlying immune-mediated inner ear disease.

The pathology of Meniere’s disease has historically been defined as an inner ear disorder presenting with endolymphatic hydrops. It is well known that some patients with Meniere’s disease show remarkable recovery from fluctuating and refractory SNHL or vertigo following systemic corticosteroid treatment; therefore, an immune-mediated mechanism has been implicated in the pathology of Meniere’s disease (Hughes et al., 1983; Derebery et al., 1991). In a study of patients with Meniere’s disease, immunohistochemistry showed IgG deposition in the endolymphatic sac obtained by surgical biopsy in 10 of 23 patients (Dornhoffer et al., 1993). Alleman reported that 3 of 30 (10%) patients with Meniere’s disease showed a positive serum reaction against proteins extracted from the endolymphatic sac of autopsy samples, and clinical data have shown an association between immunoreactivity and the disease severity (Alleman et al., 1997), suggesting an autoimmune component in some cases of Meniere’s disease. In contrast, other studies report a relationship between herpes simplex virus and the pathology of Meniere’s disease (Bergstrom et al., 1992; Kumagami, 1996). Although it appears likely that an immune reaction is involved in the pathophysiology of Meniere’s disease, the mechanism of endolymphatic hydrops, whether caused by viral infection, autoimmune reaction, or both, remains to be elucidated.

Multisystemic, organ-nonspecific autoimmune pathology may involve the inner ear, leading to secondary SNHL. A limited number of studies have evaluated human temporal bones from patients with autoimmune disease, such as Wegener granulomatosis, polyarteritis nodosa, Cogan syndrome, and lupus (McCabe, 1989; Moscicki et al., 1994). Some specimens showed fibrosis and osteoneogenesis, consistent with the end stage of inflammation. Other bones demonstrated atrophy of the stria vascularis, the organ of Corti, and the spiral ganglion without evidence of inflammation, findings consistent with ischemia. Dettmer et al. (2011) reported that the temporal bones of Crohn’s disease patients with granulomatous inner ear disease demonstrated mild chronic inflammation, poorly defined granulomas, and infiltration of CD68-positive macrophages.

Cytomegalovirus (CMV) is the leading cause of human non-hereditary congenital hearing loss. Approximately 10–20% of children with congenital CMV infection exhibit varying degrees of hearing loss (Barbi et al., 2003; Numazaki and Fujikawa, 2004). However, the pathology of congenital CMV infection within the inner ear is poorly understood. Various animal models have been employed to study the pathology of SNHL caused by intrauterine CMV infection (Woolf et al., 1989; Juanjuan et al., 2011; Wang et al., 2013). Two studies using mouse CMV infection models reported that CMV DNA was detected in spiral ganglion neurons and the stria vascularis (Juanjuan et al., 2011; Wang et al., 2013), suggesting a potential therapeutic target in CMV-induced SNHL. Multiple studies have focused on developing effective vaccines or antiviral therapy for congenital CMV infection. Unfortunately, there is no clinically effective vaccine for congenital CMV infection or CMV-induced SNHL (Arvin et al., 2004).

Several mechanisms have been postulated as the pathophysiology of sudden deafness, including microcirculatory disturbances caused by thrombosis, microtrauma or rupture of endolymph, viral infection or reactivation, and immune-mediated reaction.

One of the main pathophysiology theories of idiopathic sudden deafness is that viral infection or reactivation in the inner ear damages critical structures in the cochlea. Increased serum concentrations of antibodies against CMV, herpes zoster, herpes simplex type 1, influenza B, and mumps have been reported in patients with idiopathic sudden deafness (Merchant et al., 2008; Pyykko and Zou, 2008). Cochlear enhancement on magnetic resonance imaging (MRI) is a potential sign of inflammation in the inner ear and has been observed in some patients suffering from sudden deafness (Stokroos et al., 1998). The inner ear enhancement on MRI disappeared following resolution of hearing loss in 2 of 12 patients with sudden deafness (Mark et al., 1992). Garcia-Berrocal et al. (1997) reported a decreased concentration of both CD4^+^ and CD8^+^ cells in patients compared to healthy control subjects, suggesting an abnormal autoimmune response in lymphocyte subpopulations in patients with sudden deafness. In addition, western blot assay showed a response to recombinant human heat shock protein 70, a non-specific heat shock protein, in 19 of 58 (33%) patients with idiopathic SNHL (Tebo et al., 2006). An analysis of 11 human temporal bones from patients with sudden SNHL showed that the morphology of the stria vascularis and spiral ligament were relatively preserved, supporting a viral etiology rather than a vascular insufficiency (Linthicum et al., 2013). These findings suggest that immune mechanisms, including T cell-mediated and antibody responses, are involved, at least in part, in the onset or progression of idiopathic sudden deafness.

## EVIDENCE OF THE IMMUNE SYSTEM IN THE INNER EAR

**FIGURE 1:**
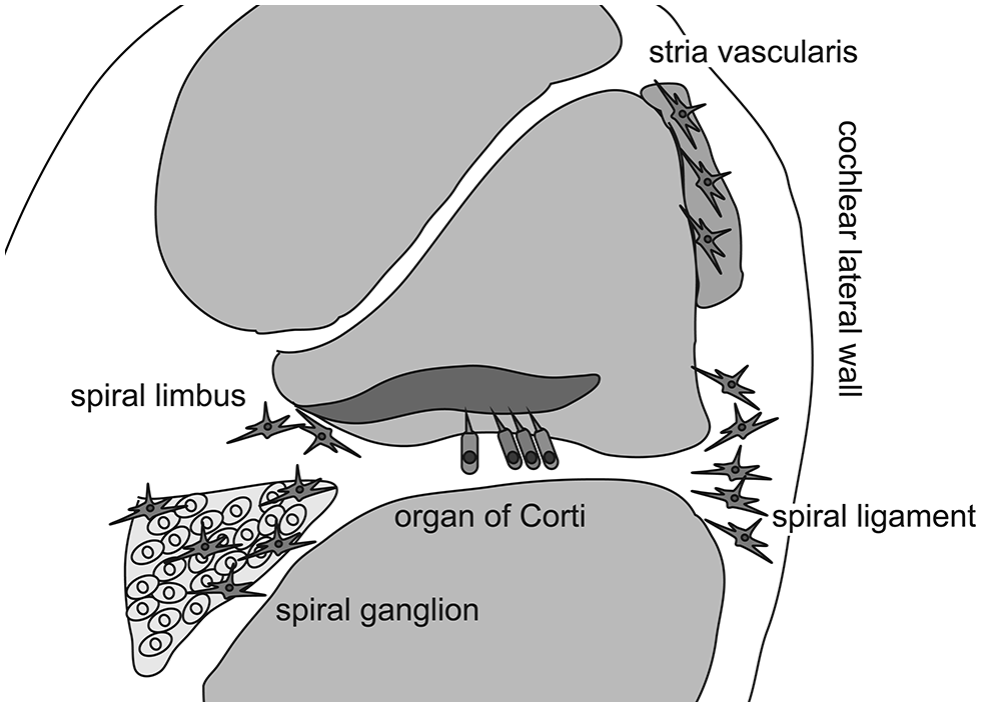
**Distribution of cochlear macrophages.** Schematic drawing shows the cross section of the cochlear duct. Cochlear macrophages reside in the spiral ligament and spiral limbus where fibrocytes are located to keep ion exchanges. In addition, macrophages are distributed in the spiral ganglion. Finally, macrophage-like melanocytes are observed in the intermediate layer of the stria vascularis.

As previously mentioned, the inner ear was believed to be “immune-privileged” and to exclude all immunocompetent cells, except in the endolymphatic sac, for a long time because chronic degeneration without neutrophilic infiltration in the organ of Corti has been described in patients with presbycusis or hearing loss due to chronic noise exposure. However, Rask-Andersen and Stahle (1979) initiated a new era of inner ear immunology by describing intimate contact between the lymphocytes and macrophages in the endolymphatic sac of guinea pigs. This association suggested that two cell types mediated the antigen-presenting process in the endolymphatic sac. The presence of immunocompetent cells and phagocytized antigen within macrophages was also reported in the endolymphatic sac (Harris et al., 1997). These findings revealed the specific role of the endolymphatic sac in antigen processing and immune activity in the inner ear. However, recent studies have demonstrated the presence of immunoreactive cells in other areas of the inner ear even under normal conditions (Lang et al., 2006; Okano et al., 2008; Sato et al., 2008). Lang et al. (2006) reported that bone marrow-derived cells of hematopoietic origin migrate into the cochlea and reside in the cochlear modiolus and the cochlear lateral wall. They also showed that bone marrow-derived cells in the cochlea express ion transporters such as the sodium/potassium/chloride co-transporter or sodium/potassium-ATPase in the cochlear lateral wall, which contains several types of fibrocytes. In a study using bone marrow-chimeric mice that were transplanted with hematopoietic stem cells after receiving lethal systemic irradiation, Okano et al. (2008) demonstrated that bone marrow-derived cells reside as macrophages in the cochlea. They also reported that Iba-1-positive macrophages were continuously and slowly replaced by bone marrow-derived cells from the systemic circulation over several months. Finally, Sato et al. (2008) reported that bone marrow-derived cells expressing CX3CR1, a fractalkine receptor specific to monocytes, natural killer cells, activated T-cells, and tissue macrophages, reside in the spiral ganglion and spiral ligament. In addition, they showed that CX3CR1-positive cells were repopulated in the cochlea over several months. Collectively, these findings indicate that the inner ear harbors immunocompetent cells of hematopoietic origin normally, with most cells likely to be tissue macrophages phenotypically. Although these tissue macrophages are distributed primarily in the spiral ganglion, spiral limbus, and spiral ligament, macrophage-like melanocytes are also observed in the intermediate layer of the stria vascularis (Zhang et al., 2012). These melanocytes reside adjacent to blood vessels and are believed to be perivascular-resident macrophages that contribute to the formation of the blood-intrastrial fluid barrier (**Figure 1**).

The role of cochlear macrophages and mechanisms of macrophage migration into the cochlea remain largely unknown. Previous studies demonstrated that injury of the auditory sensory epithelium induces inflammation characterized by macrophage infiltration into the chick basilar papilla (Warchol, 1997; Bhave et al., 1998). A large increase in the number of CD45-positive cells has been observed after noise exposure in the mouse cochlea, suggesting inflammation caused by bone marrow-derived cells (Hirose et al., 2005; Tornabene et al., 2006). The number of cochlear macrophages is also increased after aminoglycoside insult in both the spiral ganglion and spiral ligament (Sato et al., 2010). These findings indicate that cochlear macrophages play important roles in the onset and progression of inflammation after damage to the cochlear sensory epithelium. Macrophages in the cochlea are likely involved not only in the degeneration of the organ of Corti, but also the stria vascularis. Jabba et al. (2006) reported that hyperpigmentation of the stria vascularis and reorganization of marginal cells occurs in *Slc26a4*-null mice, a mouse model of Pendred syndrome, and is associated with the invasion of CD68-positive macrophages. Similar findings regarding hyperpigmentation of the stria vascularis have also been reported in genetically modified mice (Singh and Wangemann, 2008; Lu et al., 2012). The invasion of macrophages is restricted to the degenerated stria vascularis, suggesting contribution from the cochlear macrophages to degeneration or regeneration of the stria vascularis and the cochlear lateral wall.

The number of cochlear macrophages is also increased by systemic administration of macrophage colony stimulating factor (Csf1), which is one of the primary regulators of mononuclear phagocyte activation. The density of Iba1-positive macrophages is increased in both the spiral ligament and spiral ganglion 1 day after administering Csf1 (Okano et al., 2008), but it is unclear whether the increased macrophage population is due to migration from the circulation or *in situ* proliferation in the cochlea. Yagihashi et al. (2005) also demonstrated that topical administration of Csf1 ameliorates the degradation of auditory neurons following surgical injury in a rat model. In addition, Csf1 was demonstrated to have neuroprotective properties in an *in vitro* model of excitotoxicity in hippocampal neurons, suggesting both direct and indirect effects of Csf1 on survival of targeted cells (Vincent et al., 2002). It is unknown whether the activation of tissue macrophages has protective or degenerative effects in the target organ, but control of macrophage activity through Csf1 administration is a potential approach for several inner ear disorders.

Previous reports investigating *in situ* proliferation of cochlear macrophages are controversial. Using Bromodeoxyuridine labeling, Hirose et al. (2005) reported that cochlear macrophages do not proliferate after acoustic trauma. However, according to the study done by Okano et al. (2008) a subset of macrophages expressed Ki67, suggesting that resident macrophages enter the cell cycle after migration following surgical invasion of the cochlea. Although the precise nature of migrating macrophages is to be determined, cochlear macrophages are most likely responsible for several different inner ear pathologies.

## TISSUE MACROPHAGES IN THE INNER EAR

In general, adaptive immune cells play a major role in disease progression, and the innate immune system, primarily monocytes and macrophages, plays a central role in the onset of immune activity. The concept of multiple macrophage activation states is not new, but extending this idea to resident tissue macrophages has garnered increased interest in recent years. Unfortunately, research of peripheral macrophage polarization might not accurately describe their central nervous system counterparts.

Macrophages are derived from monocyte precursors that undergo tissue-specific differentiation and infiltrate the site of infection or injury to produce inflammatory mediators. The cells typically polarize into the pro-inflammatory M1 phenotype and function as an effector of the Th1-mediated immune response. The M1 polarization of macrophages is regulated by several factors including the mineralocorticoid receptor (Lawrence and Natoli, 2011). In the normal course of inflammation, the immune process is controlled, and M1-macrophages undergo apoptosis or switch to the anti-inflammatory M2 phenotype, thereby halting inflammation. However, if the inflammatory response of macrophages is not controlled, it becomes pathogenic, resulting in significant levels of non-specific tissue damage and leading to inflammatory and autoimmune diseases (Wynn et al., 2013). Therefore, macrophage-targeted therapy is extremely relevant in improving the prognosis of inflammatory diseases, particularly inflammation in the inner ear.

Thought provoking observations have been obtained in studies of patients with human immunodeficiency virus (HIV), specifically concerning macrophage function in the inner ear. Monocytes and macrophages are susceptible to HIV infection and are considered a main mechanism responsible for central nervous system infection in areas containing perivascular macrophages and parenchymal microglia (Burdo et al., 2013). Lin et al. (2013) demonstrated that HIV infection is significantly associated with an increased risk of developing sudden deafness in patients aged between 18 and 35 years. In addition, Assuiti et al. (2013) found no direct association between anti-retroviral therapy and hearing loss but stressed the need for future investigation of the causes and association between anti-retroviral therapy and hearing loss. These data suggest that deficiencies in the macrophage and monocyte lineage may lead to dysfunction in the inner ear and highlight the important roles of macrophages in the maintenance of auditory function.

Several surface markers have been used in the animal studies of macrophages to immunohistochemically test their phenotypes and distribution in the tissues. CD68 is a heavily glycosylated transmembrane protein and is a common surface marker expressed in all macrophages (Smith and Koch, 1987; Ramprasad et al., 1996). F4/80 is a member of a gene family that includes the human epidermal growth factor module-containing mucin-like hormone receptor 1 and human CD97, and resides on the surface of a family of cells that includes all well differentiated members of the mononuclear phagocyte system. Although the precise function of F4/80 is not completely understood as F4/80-null mice have no remarkable phenotype, F4/80-positive cells have many common features regardless of their tissue location and are characterized by highly ramified cell shape (Hume et al., 2002). Iba1 is a calcium binding protein specific to macrophages that mediates calcium signals that may control migration and phagocytosis in tissue macrophages (Imai et al., 1996). Reportedly, tissue macrophages in the inner ear express Iba1 in addition to F4/80 (Okano et al., 2008). Csf1r is an alternative surface marker on macrophages and is thought to play key roles in the proliferation, differentiation, and survival of macrophages (Hume et al., 2002). In other categorical systems, the differentiation of monocytes and macrophages is described based on the expression of specific cell markers. If similar markers could be identified in tissue macrophages or cells of monocyte lineage, it may be possible to trace these cells along several different points of the inner ear pathophysiology, including systemically circulating monocytes, migrating monocytes, and resident tissue macrophages.

## CORTICOSTEROID THERAPY

Systemic or possibly local administration of corticosteroids is the mainstay of treatment for SNHL, including immune-mediated inner ear disease. However, there are limited prospective data evaluating the appropriate dose, route, and length of corticosteroid treatment. In addition, although many patients experience a short-term response to steroids, the response is generally not sustained (Zeitoun et al., 2005). A prospective, randomized, controlled study in 116 patients with rapidly progressive, bilateral SNHL reported that 57% of patients in the 1 month prednisone challenge showed improved hearing, but adverse effects such as hyperglycemia were observed in 14% of patients (Alexander et al., 2009). A meta-analysis of the management of idiopathic sudden SNHL performed by Spear and Schwartz (2011) reported that intratympanic corticosteroids administered as the primary treatment appeared equivalent to treatment with high-dose oral prednisone. Furthermore, intratympanic administration of corticosteroids potentially recovered some degree of hearing as a salvage therapy. These observations suggest that the local administration of corticosteroids is beneficial through mechanisms distinct from those of systemic corticosteroid therapy.

Despite numerous clinical reports of corticosteroid treatment for SNHL, the spontaneous rate of recovery in acute SNHL complicates conclusions about corticosteroid efficacy. To date, the mechanisms underlying fluctuating SNHL in an immune-mediated inner ear disease are unclear. We know little on how corticosteroids work in the inner ear and which parts of the inner ear are affected during reversible hearing loss. The expression of glucocorticoid receptors in the inner ear is limited to the inner and outer hair cells, the spiral ganglion, and the spiral ligament (Tahera et al., 2006; Meltser et al., 2009). In addition to glucocorticoid receptors, corticosteroids have a strong affinity for mineralocorticoid receptors. The use of systemic mineralocorticoids alone or in combination with glucocorticoids has not been evaluated in humans, but is apparently efficacious in animal models (MacArthur et al., 2008). Because the inner ear requires tight regulation of ion homeostasis in both the perilymph and endolymph, the effect of corticosteroid therapy through mineralocorticoid receptors should be considered in the mechanism of action when treating SNHL. Moreover, there are several questions on the assumptions which clinicians and researchers take for granted. Do corticosteroids only suppress inflammation and immune response in the inner ear? Do corticosteroids affect the inner ear specifically or do the systemic effects of corticosteroids benefit the inner ear disorder? Does immune-mediated hearing loss always respond to corticosteroids? A better understanding of the immune-mediated aspects of hearing loss and specific diagnostic assays would lead to the development of immune-modulating therapy for sudden or progressive SNHL.

McCabe recommended high-dose corticosteroids along with cyclophosphamide therapy for prolonged treatment of immune-mediated inner ear disease (McCabe, 1979). However, the extended follow-up of patients treated with cyclophosphamide revealed potential adverse effects and long-term morbidity and mortality risks of the agent, particularly neoplasm development in younger patients, which limited its use and prompted the search for other immunosuppressive options (Harris et al., 2003; Garcia-Berrocal et al., 2006).

Methotrexate has been used as a sparing treatment to control refractory immune-mediated SNHL. Salley et al. (2001) reported improvement in the majority of 53 patients with immune-mediated inner ear diseases who were treated with low-dose methotrexate. Long-term, low-dose methotrexate therapy appeared to be effective in at least some patients with immune-mediated hearing loss that is refractory to traditional corticosteroid therapy (Matteson et al., 2001). By contrast, a randomized, double-blind, placebo-controlled trial in 2003 of immune-mediated inner ear disease suggested that methotrexate does not appear to be effective in maintaining the hearing improvement achieved with prednisone therapy (Harris et al., 2003).

Azathioprine was also reported as an alternative option in treating immune-mediated inner ear disease, although reports were based on small case series and were inconclusive (Lasak et al., 2001).

According to these findings, systemic immunosuppressives such as methotrexate are effective in some patients with bilateral, progressive, or fluctuating SNHL, which indicates an immune component in the pathophysiology of hearing loss. However, the diagnostic criteria of immune-mediated inner ear disease vary among previous reports. Clinicians and investigators should consider that patients with bilateral fluctuating SNHL do not always have an immune disorder in the inner ear.

## RECENT ADVANCES AND FUTURE DIRECTIONS OF SNHL TREATMENT

### MOLECULAR-TARGETED DRUGS AND BIOLOGICAL AGENTS

Despite initial optimistic reports suggesting a therapeutic effect of methotrexate, a recent study by Harris et al. (2003) failed to demonstrate its efficacy for long-term management of immune-mediated inner ear diseases as mentioned above. Instead, molecular-targeted drugs have garnered attention of investigators and clinicians in the fields of immunology and audiology due to their specificity against therapeutic targets, resulting in less toxicity and fewer adverse effects.

Etanercept is a fusion protein comprising two recombinant tumor necrosis factor (TNF) receptors linked to the C portion of human IgG1 (Mohler et al., 1993). A retrospective case series by Rahman et al. (2001) examined the response to etanercept in 12 patients with immune-mediated haring loss responsive to high-doses of corticosteroids. Improvement or stabilization of hearing and tinnitus was observed in 91% of patients, suggesting that etanercept therapy is safe and may be efficacious in some patients with immune-mediated hearing loss. By contrast, two studies reported that etanercept has no substantial efficacy in improving hearing loss (Cohen et al., 2005; Matteson et al., 2005). Further studies are needed evaluating alternative regimens that use etanercept or other anti-TNF-alpha agents.

Infliximab is another monoclonal antibody against TNF-alpha that binds TNF-alpha and reduces its activity (Siddiqui and Scott, 2005). A retrospective review of eight patients with suspected immune-mediated hearing loss refractory to conventional treatment examined the efficacy of infliximab on hearing improvement; however, none of the patients exhibited a positive response to infliximab therapy based on objective measurements (Liu et al., 2011). Monoclonal antibody therapy directly targeting cells in the inner ear is unlikely to be effective because the concentration of immunoglobulin is much lower in this region than that in cerebrospinal fluid or blood due to tight regulation by the blood-labyrinthine barrier. Accordingly, transtympanic administration of infliximab was evaluated by Van Wijk et al. (2006) in nine patients with immune-mediated hearing loss. Transtympanic administration of infliximab resulted in hearing improvement and reduced disease relapses, indicating the potential utility of local administration of monoclonal antibody in treating inner ear disease.

Adalimumab was also used to block TNF signaling in patients with immune-mediated hearing loss, but reports were based on a small number of cases (Morovic Vergles et al., 2010).

Rituximab is a genetically engineered chimeric monoclonal antibody against CD20, which resides the surface of B cells. The agent reduces autoantibody production both in circulating and tissue B cells, but does not affect plasma cells. A small pilot study in patients with immune-mediated inner ear diseases was performed evaluating the efficacy of rituximab in treating hearing loss (Cohen et al., 2011). Further evaluation of rituximab is encouraged using a properly designed randomized study.

### NUCLEIC ACID THERAPY

Nucleic acid therapy, including delivery of gene constructs to increase or force expression in the targeted tissue, and small interfering RNA to block expression of a specific gene, is a promising approach for treating inner ear disease. However, limited access to the lesion site creates challenges in nucleic acid therapy of the inner ear. Various studies employing animal models utilize viral vectors to introduce the nucleic acid into the inner ear, but there are toxicity and safety concerns associated with this method, including immunogenicity and mutagenesis. Non-viral vectors are advantaged by overcoming these limitations plaguing viral vectors. Although nucleic acid therapy is challenging in the *in vivo* setting, the development of novel delivery systems could lead to drastic advances in improving the prognosis of patients with SNHL. Obviously, macrophages are a potential target for nucleic acid therapy using novel delivery systems in the inner ear, controlling not only inflammation and degeneration of sensory organs, but also regeneration of the cochlear lateral wall and innervation from the spiral ganglion neurons to hair cells.

### DELIVERY OF GENE MODIFIED MACROPHAGES

**FIGURE 2:**
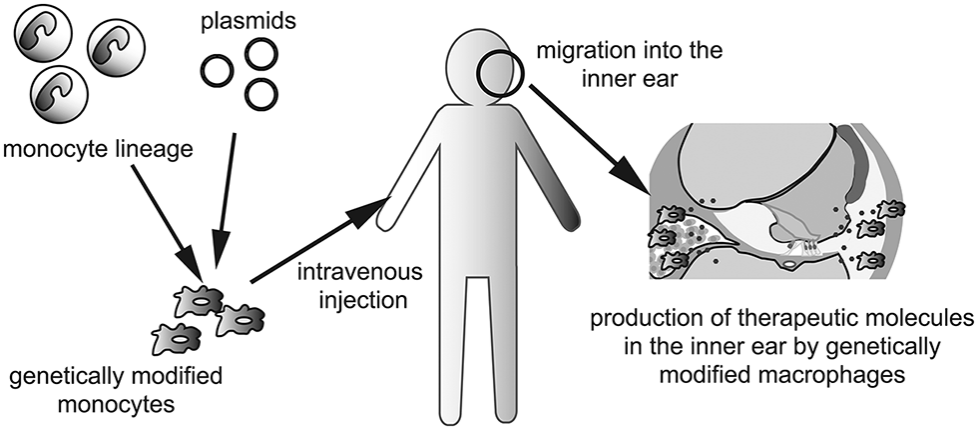
**Schematic depictions of a concept to utilize genetically modified macrophages for the treatment of inner ear diseases.** Autologous monocyte lineage is taken from patient’s peripheral blood, and transfected plasmids of targeted gene. After *ex vivo* culturing, genetically modified monocytes are injected intravenously. Injected monocytes are migrating into the inner ear to differentiate to tissue macrophages. Genetically modified macrophages produce and secrete therapeutic molecules which diffuse throughout the fluid of the inner ear.

The last, but not least, the use of genetically modified monocytes or macrophages as vectors should be considered for production of therapeutic molecules or factors that promote regeneration or regrowth of specific structures in the inner ear. This concept is especially well suited for a secreted paracrine or endocrine factor such as a hormone or growth factor. Because the inner ear contains three fluid-filled compartments, secreted factors from genetically modified macrophages could potentially diffuse throughout the inner ear without help from the blood or lymphatic circulation. Although the use of genetically modified cells as vectors of genes or pharmacotherapeutic reagents is in the early stage (Hakuba et al., 2005; Okano et al., 2006; Kesser and Lalwani, 2009), transplantation of genetically engineered cells able to secrete specific metabolic or humoral cues could augment pharmacologic immune modulation in the inner ear. Delivery of genetically modified cells into the inner ear could pose a major challenge because of the anatomical characteristics of the inner ear. Monocytes and macrophages are able to migrate into the inner ear in both pathologic and normal conditions (Hirose et al., 2005; Okano et al., 2008). Thus, the human monocyte lineage could be isolated and cultured *ex vivo* and genetically manipulated. Intravenous administration of genetically modified monocytes could enable them to reach and migrate into the inner ear, although tissue- or organ specificity could be a potential problem to overcome in clinical applications (**Figure 2**).

Apart from resident macrophages at the disease site, circulating monocytes are continuously recruited to meet the demands of the inflammatory response and the expression of chemokines, cytokines, and cell adhesion molecules. An alternative approach is to facilitate phagocytosis of loaded delivery vehicles by monocytes, which then passively targets the site of disease due to the mounting immune response. The active targeting approach is most attractive and promising if the surface of the delivery vehicle can be decorated with a ligand that selectively interacts with their target receptors. Further research evaluating the use of monocytes as vehicles is desired.

## CONCLUSION

In this review, we discussed the involvement of the immune system in the pathology of SNHL, particularly the innate immune system in the inner ear and the pathology of immune-mediated inner ear disease. Recent advances in basic and clinical audiology and immunology research has been rapid. Although there is still much work to be done, we believe that the future of inner ear immunology and SNHL treatment are bright and promising.

## Conflict of Interest Statement

The author declares that the research was conducted in the absence of any commercial or financial relationships that could be construed as a potential conflict of interest.
